# Sacral Chordoma: Multimodality Imaging and Radiologic-Pathologic Correlation of a Rare Malignant Sacral Tumor

**DOI:** 10.7759/cureus.108491

**Published:** 2026-05-08

**Authors:** Vinutha Honneshaiah, Kavya Birapur, Mohit Mouna R

**Affiliations:** 1 Radiology, Sri Siddhartha Academy of Higher Education, Tumkur, IND; 2 Radiology, Sri Siddhartha Institute of Medical Sciences, Bengaluru, IND; 3 Radiology, East Point Medical College, Bengaluru, IND

**Keywords:** magnetic resonance imaging, presacral mass, radiologic-pathologic correlation, sacral chordoma, sacral tumor

## Abstract

Chordoma is a rare malignant tumor arising from embryonic notochordal remnants and most commonly involves the sacrococcygeal region. Because of its slow-growing nature and nonspecific clinical presentation, diagnosis is often delayed until significant local extension occurs. Imaging plays a crucial role in lesion detection, characterization, and surgical planning. We report a case of sacral chordoma in a 63-year-old woman presenting with chronic sacral pain and constipation. Initial radiographs were subtle, demonstrating non-visualization of the distal sacral segments and ill-defined sacral soft tissue fullness. CT revealed a large destructive lytic lesion involving the sacrum from S2 to the distal sacrococcygeal segments with internal chunky calcifications, sacral canal involvement, and bulky presacral extension. MRI demonstrated a lobulated mass with iso- to hypointense T1 signal, heterogeneous T2/short TI inversion recovery (STIR) hyperintensity, diffusion restriction, and avid heterogeneous enhancement with central non-enhancing areas. Histopathology confirmed chordoma with characteristic physaliphorous cells embedded in a myxoid stroma. This case highlights the characteristic multimodality imaging appearance of sacral chordoma and emphasizes the importance of radiologic-pathologic correlation in establishing diagnosis and narrowing the differential considerations.

## Introduction

Sacral tumors are uncommon lesions that often present with nonspecific symptoms, resulting in delayed diagnosis and substantial local disease at presentation. Imaging, therefore, plays a crucial role in lesion detection, characterization, differential diagnosis, and surgical planning. Familiarity with the characteristic radiologic appearance of malignant sacral tumors is important for both clinicians and radiologists.

Chordoma is a rare malignant neoplasm arising from persistent embryologic remnants of the primitive notochord and accounts for approximately 1%-4% of primary malignant bone tumors [1,2]. These tumors are slow-growing but locally aggressive, often producing progressive osseous destruction and soft tissue extension. Chordomas predominantly involve the axial skeleton, with the sacrococcygeal region representing the most common site of occurrence, followed by the clivus and mobile spine [3,4]. Sacral chordomas typically arise from the lower sacral segments and demonstrate a characteristic midline location related to the embryologic distribution of notochordal remnants.

Clinical presentation is frequently delayed because of the indolent nature of the lesion and deep pelvic location. Patients commonly present during the fifth to seventh decades of life, with nonspecific symptoms such as chronic sacral pain, constipation, bladder dysfunction, or lower extremity neurologic symptoms related to neural foraminal involvement [5,6]. Chronic pain that worsens while sitting is a common presenting feature and may reflect increasing local mass effect.

Multimodality imaging plays a central role in diagnosis, differential characterization, and surgical planning. Radiographs may be subtle or inconclusive, whereas CT is valuable for evaluating osseous destruction, cortical breach, and intratumoral calcifications. MRI provides superior characterization of marrow involvement, neural foraminal extension, and adjacent soft tissue spread [3-5]. Typical MRI findings include low-to-intermediate T1 signal intensity, marked T2 hyperintensity related to abundant myxoid matrix, and heterogeneous enhancement following contrast administration [5,7]. Histopathologically, chordomas are characterized by cords and nests of tumor cells embedded within a myxoid stroma, with physaliphorous cells representing a hallmark diagnostic feature [2].

We present a case of sacral chordoma in a 63-year-old woman with chronic sacral pain and constipation, emphasizing the characteristic multimodality imaging findings and radiologic-pathologic correlation.

## Case presentation

A 63-year-old woman presented with gradually progressive sacral pain for six months, which worsened while sitting. She also reported associated constipation during the same period. There was no history of trauma, fever, weight loss, bowel or bladder incontinence, or known malignancy. Neurological examination revealed no motor weakness or sensory deficits, and laboratory investigations were within normal limits.

**Figure 1:**
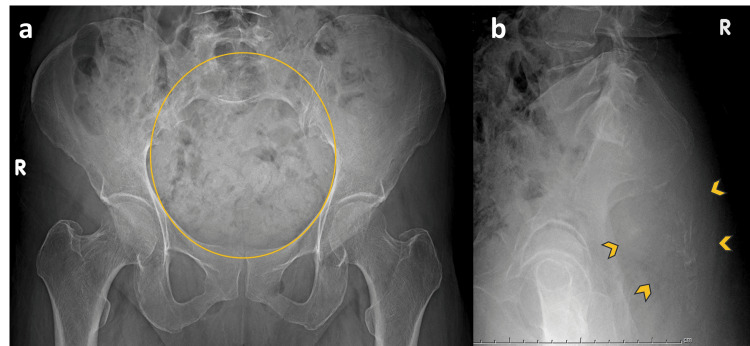
Subtle radiographic findings of sacral chordoma Anteroposterior (a) and lateral (b) pelvic radiographs demonstrate non-visualization of the distal sacral segments (circled area) with subtle loss of normal sacral contour. Ill-defined soft tissue fullness is seen in the sacral and presacral region (arrowheads). Findings are inconspicuous, highlighting the limited sensitivity of plain radiography.

Plain radiographs of the pelvis were relatively inconspicuous. The anteroposterior view demonstrated non-visualization of the distal sacral segments with subtle loss of normal sacral contour, while the lateral radiograph showed ill-defined soft tissue fullness in the sacral and presacral region, raising suspicion for an underlying mass. No aggressive periosteal reaction was identified (Figure 1).

**Figure 2:**
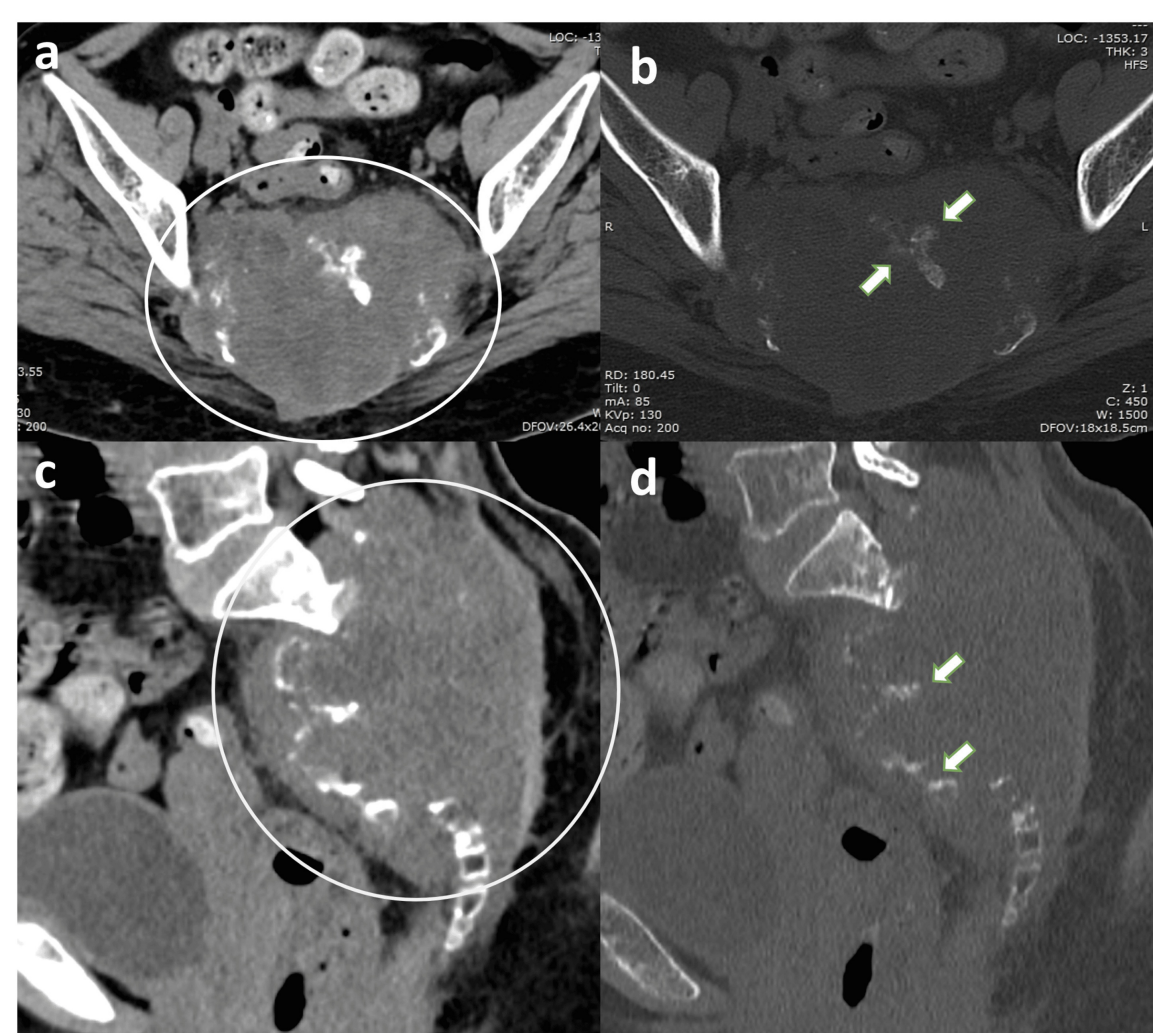
CT demonstration of sacral destruction and internal calcifications Axial and sagittal reformatted CT images in soft tissue (a, c) and bone windows (b, d) demonstrate a lobulated destructive lytic lesion centered within the sacrum (circled area). Extensive cortical destruction and expansile remodeling are present, with multiple internal calcific foci (arrows) representing matrix mineralization.

Subsequent CT imaging revealed a large lobulated expansile destructive lytic lesion involving the sacrum from the S2 vertebral level to the distal sacrococcygeal segments. Extensive osseous destruction with cortical breach was present, accompanied by a bulky soft tissue component. Multiple chunky internal calcifications were identified within the lesion. The mass completely filled the sacral canal with obliteration of the involved sacral neural foramina and extended laterally into both sacroiliac joints. Anteriorly, it formed a large presacral soft tissue component causing mass effect on the rectum (Figure 2).

**Figure 3:**
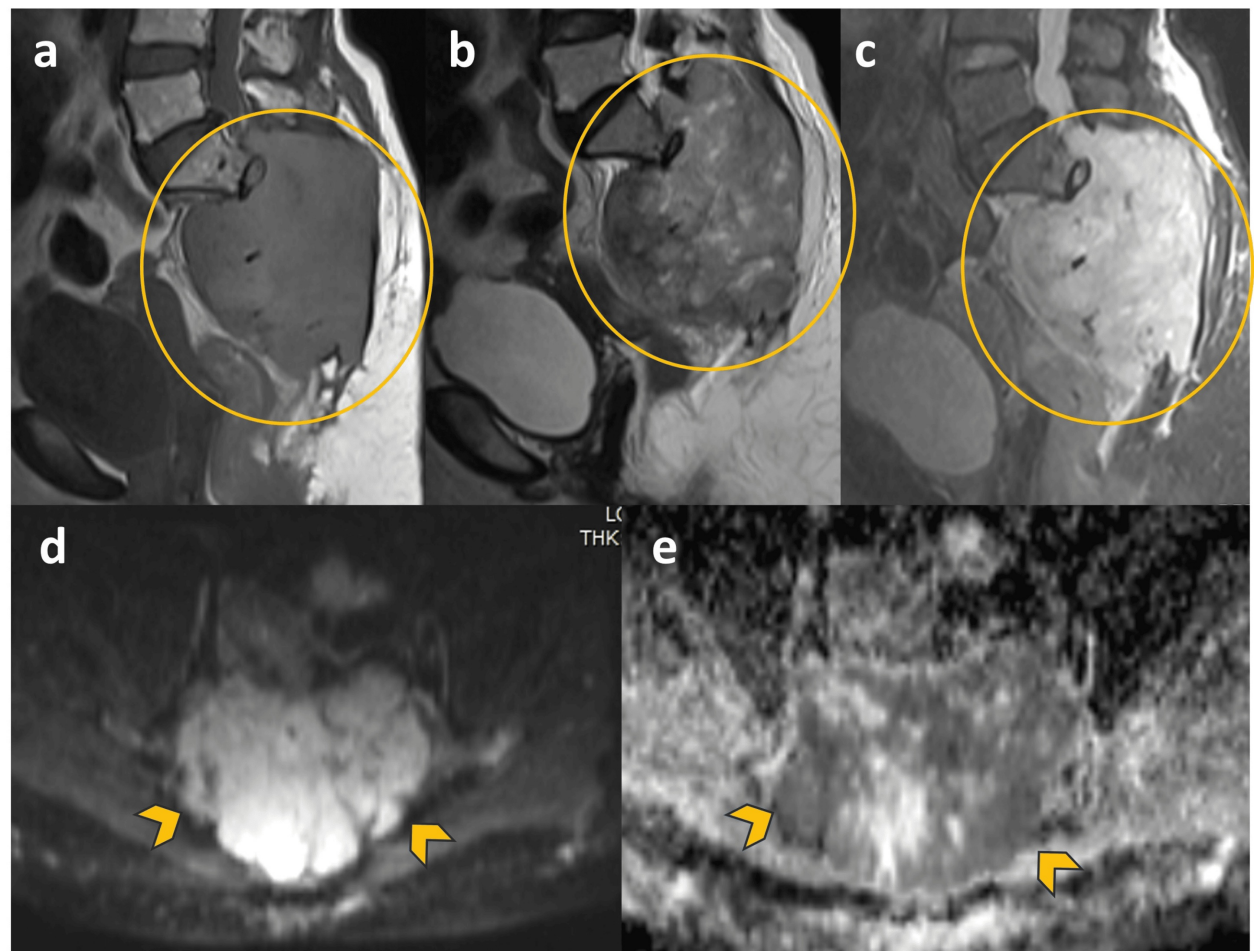
MRI signal characteristics of sacral chordoma MRI demonstrates a lobulated sacral mass with iso- to hypointense signal on T1-weighted imaging (a) and heterogeneous hyperintensity on T2-weighted and STIR sequences (b, c). The lesion fills the sacral canal and extends into the presacral space (circled area). Diffusion-weighted imaging (d) demonstrates moderate restricted diffusion with corresponding low signal on ADC mapping (e) (arrowheads).

**Figure 4:**
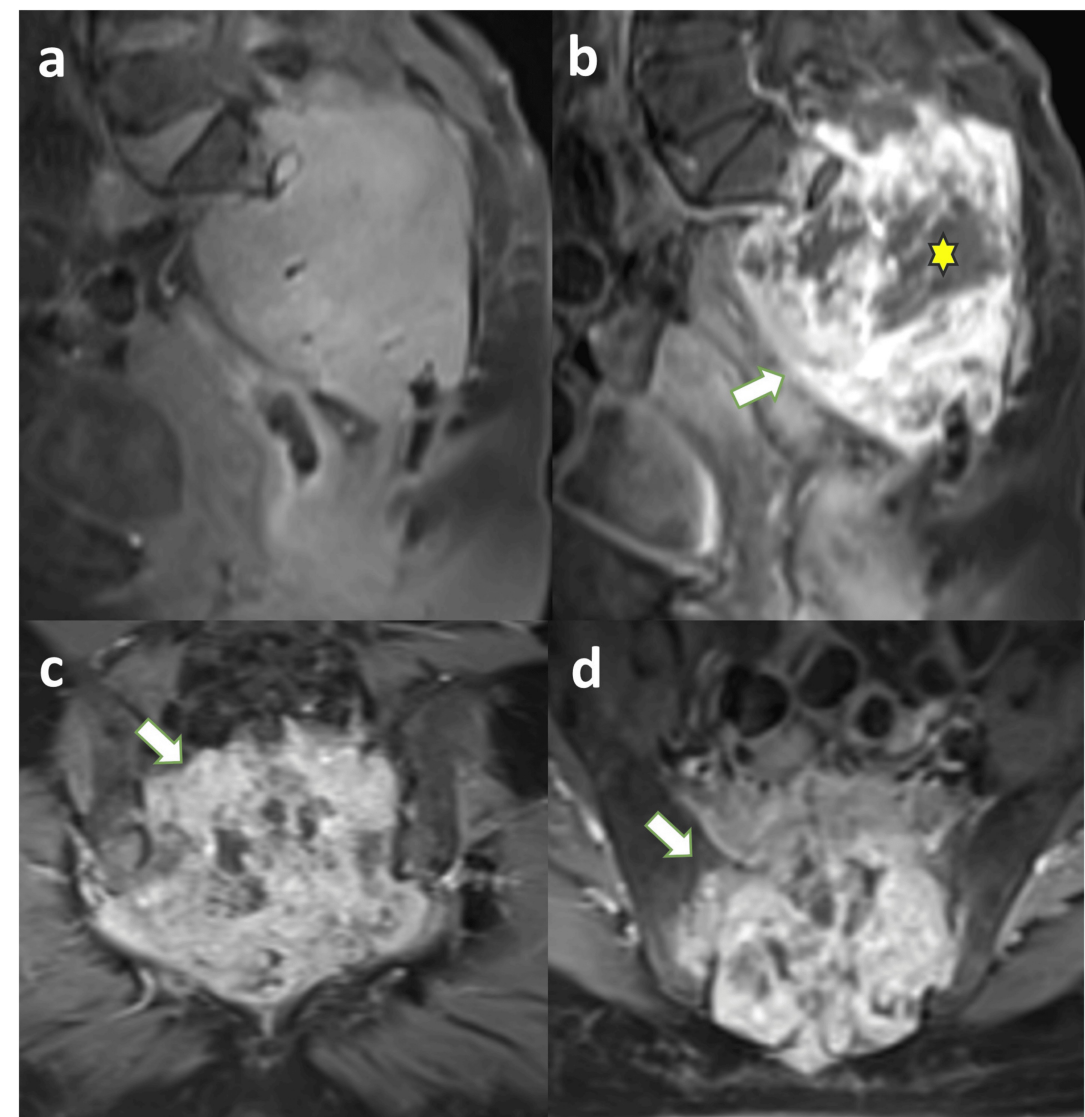
Contrast-enhanced MRI showing heterogeneous tumor enhancement Pre-contrast (a) and post-contrast fat-suppressed T1-weighted MRI images (b-d) demonstrate avid heterogeneous enhancement of the sacral soft tissue mass (arrows). Central non-enhancing regions (asterisk) correspond to necrotic or myxoid components within the lesion.

MRI demonstrated a lobulated sacral mass measuring approximately 9.8 × 8.2 × 11 cm (anteroposterior × transverse × craniocaudal dimensions). The lesion appeared predominantly iso- to hypointense on T1-weighted images and heterogeneously hyperintense on T2-weighted and short TI inversion recovery (STIR) sequences, reflecting high fluid and myxoid content. Diffusion-weighted imaging showed heterogeneous areas of moderate diffusion restriction. The mass completely filled the sacral canal with obliteration of the sacral neural foramina, extended into the inferior aspects of both sacroiliac joints, and formed a large presacral component exerting mass effect on the rectum. Adjacent mesorectal fat showed inflammatory stranding, with no direct involvement of the rectum (Figure 3). Following contrast administration, the lesion demonstrated avid heterogeneous enhancement with central non-enhancing regions suggestive of necrotic or myxoid components (Figure 4).

**Figure 5:**
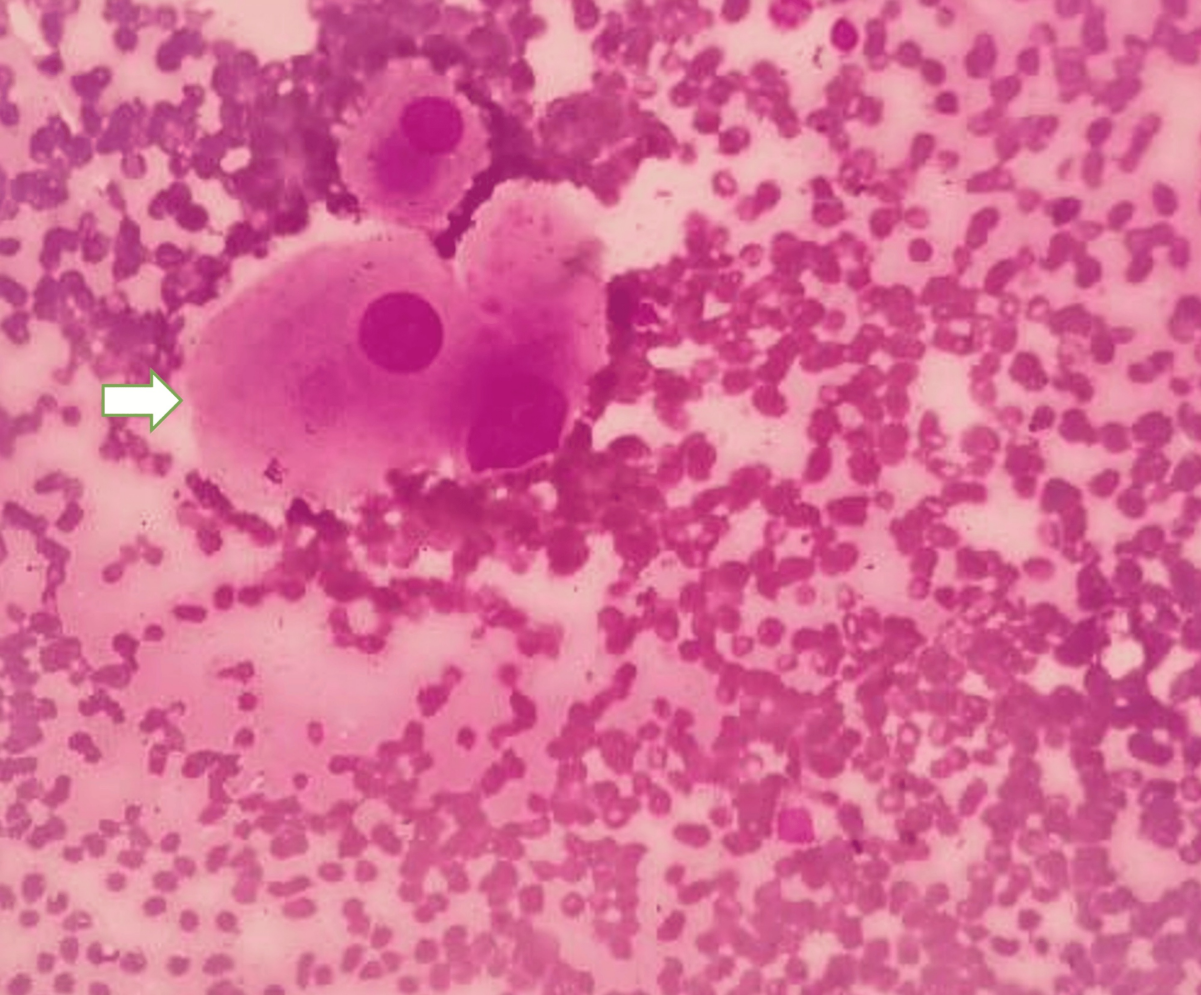
Histopathologic appearance of sacral chordoma Histopathologic image demonstrating characteristic physaliphorous cells (arrow), consistent with chordoma.

Histopathological examination revealed tumor cells arranged in cords and nests within a myxoid matrix. Large vacuolated physaliphorous cells with abundant eosinophilic cytoplasm were identified, confirming the diagnosis of chordoma (Figure 5).

## Discussion

Chordoma is a rare malignant tumor derived from embryonic notochordal remnants and represents the most common primary malignant neoplasm of the sacrum [1,2]. Because of its slow growth and deep pelvic location, diagnosis is often delayed until symptoms related to local mass effect develop. Patients commonly present with chronic sacral pain, constipation, bladder dysfunction, or neurologic symptoms secondary to sacral canal involvement [3,4]. In the present case, sacral pain and constipation were the predominant presenting complaints.

Radiographs are frequently subtle or inconclusive in sacral chordoma because of overlying bowel gas and complex pelvic anatomy. Findings may include sacral osteolysis, cortical irregularity, or presacral soft tissue fullness [3]. In this patient, plain radiographs demonstrated distal sacral non-visualization with vague presacral soft tissue fullness, emphasizing the limited sensitivity of radiography in early lesion detection.

CT is valuable for assessing osseous destruction, cortical breach, and internal calcification. Sacral chordomas typically appear as centrally located destructive lytic lesions with associated soft tissue masses [4,5]. In this case, CT demonstrated extensive sacral destruction with chunky calcifications, sacral canal involvement, and bilateral sacroiliac joint extension.

MRI provides superior delineation of lesion extent and adjacent soft tissue involvement. Typical findings include low-to-intermediate T1 signal intensity, marked T2 hyperintensity, and heterogeneous enhancement following contrast administration [5-7]. MRI also allows accurate evaluation of sacral canal invasion, neural foraminal involvement, and presacral extension. In the present case, MRI demonstrated extensive sacral canal obliteration, bilateral sacroiliac extension, and mass effect on the rectum.

The differential diagnosis of destructive sacral lesions includes giant cell tumor, chondrosarcoma, metastasis, plasmacytoma, and lymphoma [5,7]. Giant cell tumors usually occur in younger adults and often demonstrate expansile osteolytic morphology with relatively lower T2 signal intensity compared with chordoma. Chondrosarcomas typically arise eccentrically near the sacroiliac region and frequently contain characteristic ring-and-arc chondroid matrix calcifications. Metastases and plasmacytoma may demonstrate multifocal involvement and usually lack the marked T2 hyperintensity and lobulated midline morphology characteristic of chordoma. Lymphoma commonly demonstrates permeative osseous destruction with associated soft tissue components but may preserve cortical bone relative to lesion size. Recognition of a large midline sacral lesion with extensive osseous destruction, marked T2 hyperintensity, soft tissue extension, and internal calcifications strongly favors the diagnosis of chordoma [5,7].

Histopathologic confirmation remains essential. Physaliphorous cells within a myxoid stroma are characteristic findings and establish the diagnosis [2]. Surgical excision remains the primary treatment modality and is frequently combined with radiotherapy for local disease control [6,8]. Prognosis is largely influenced by local recurrence rather than distant metastasis, emphasizing the importance of accurate imaging evaluation and early diagnosis [9-11].

## Conclusions

Sacral chordoma is a rare malignant tumor that should be considered in the differential diagnosis of destructive midline sacral lesions in older adults presenting with chronic sacral pain and pelvic symptoms. Because radiographic findings may be subtle, cross-sectional imaging plays a key role in lesion detection and characterization. CT is valuable for demonstrating osseous destruction and internal calcifications, while MRI provides superior assessment of marrow involvement, neural extension, and presacral soft tissue spread. Recognition of characteristic imaging features, combined with histopathologic confirmation, is essential for accurate diagnosis and treatment planning. Multimodality imaging and radiologic-pathologic correlation remain crucial for defining disease extent and narrowing the differential diagnosis.
